# Responsivity and relation to depressive symptoms of occupational behavior and experience patterns

**DOI:** 10.3389/fpubh.2023.1271486

**Published:** 2023-12-07

**Authors:** Lilia Papst, Christian Zickwolf, Michael Käfer, Volker Beierlein, Volker Köllner

**Affiliations:** ^1^Psychosomatic Rehabilitation Research Group, Department of Psychosomatic Medicine, Center for Internal Medicine and Dermatology Charité – University Medicine Berlin, Berlin, Germany; ^2^Mediclin Bliestal Clinics, Clinic for Psychosomatic Medicine, Blieskastel, Germany; ^3^Department of Medical Psychology, University Medical Center Hamburg-Eppendorf, Hamburg, Germany; ^4^Department of Psychosomatics and Behavioural Psychotherapy, Rehabilitation Centre Seehof, Teltow, Germany

**Keywords:** depression, burnout, psychological, occupational health, occupational stress, working style

## Abstract

**Introduction:**

Work stress is a frequent factor in the development of depression. However, not only workplace environment, but also personal attitudes may affect stress experience. The aim of this study was to investigate the change sensitivity of occupational attitudes in psychosomatic inpatients and assess the relationship of changes to depressive symptom reduction.

**Methods:**

The data set encompassed *N* = 1708 inpatients from two German psychosomatic rehabilitation clinics at admission and discharge. Responsivity of AVEM measures was evaluated by Bonferroni-corrected t-tests and Cohen’s d_z_ effect sizes for paired samples. The relation of occupational behavior and experience patterns and depressive symptoms as assessed by the BDI-II questionnaire was calculated by Pearson correlation analysis of pre-post differential values.

**Results:**

Changes in work attitudes were found on eight out of eleven AVEM subscales (*P_adj_* ≤ 0.001, Cohen’s *d_z_* = −0.45 to 0.43) and all AVEM coping styles. Most patients (57.4%) were classified to have a Burnout occupational coping style at admission. Changes following rehabilitation were most frequently observed from Burnout to Sparing coping styles (8.3%). Small to moderate associations between changes in occupational attitudes and depressive symptom reduction were found for all subscales (*r* = −0.39 to 0.25) except work ambition, and for occupational coping styles Burnout (*r* = 0.19), Sparing (*r* = −0.18) and Healthy (*r* = −0.10), but not Ambitious.

**Discussion:**

The data support responsivity of occupational behavior and experience patterns within a psychosomatic rehabilitation setting. Correlations with depressive symptom reduction suggest that occupational attitudes are related but separate treatment targets.

## 1 Introduction

Work is the most frequently named source of stress in the general population in Germany (1). This poses a substantial public health issue as the presence of work-related stress increases the probability of a psychological diagnosis by two- to fourfold (2, 3), with depressive disorders being the most common diagnosis (4). Depressive disorders are associated with high personal as well as public costs. Among all diseases and all age groups, depression ranks 13th among the top causes of disease-adjusted life years globally (5). With a pooled life-time prevalence of a suicide attempt of 31%, it is associated with considerable mortality (6). Moreover, utilization of healthcare resources is increased in depressed individuals compared to the general population in Europe (RR = 5.4), as is the relative risk of work impairment (RR = 2.2) (7) and prolonged periods of sick leave (8).

Crucially, we distinguish two major aspects of work-related stress. On the one hand, there are external factors such as high workload, long working hours, lack of control, or job insecurity (9), i.e., factors describing objective aspects of occupational stress load. On the other hand, individual factors such as perfectionism (10, 11), over-commitment (12), or low self-efficacy (13, 14) may equally contribute to stress and depressive symptoms. Stress can therefore be understood as a transactional construct in that its experience is as much determined by the individual appraisal of a stressor – led by internalized beliefs and attitudes – as it is by the stressor itself (15).

The Assessment of Work-related Behavior and Experience Patterns questionnaire (AVEM) (16) allows for the evaluation of the individual’s beliefs and attitudes towards work. It distinguishes between Healthy (Type G), Sparing (Type S), Ambitious (Type A), and Burnout (Type B) patterns. The Healthy pattern is an indication of a healthy relationship towards work. It is characterized by strong but not excessive professional commitment, high resilience to stress and positive emotions. Individuals with a Sparing pattern report low work engagement but otherwise little issues in the other areas. The Ambitious pattern is marked by high effort and excessive job commitment that have no equivalent in life satisfaction. Finally, workers with a Burnout pattern experience being permanently overwhelmed, exhausted, and resigned. They are characterized by low work engagement, a lack in resistance to stress and strongly negative emotions. Previous investigations found that the Burnout pattern was positively associated with depressive symptoms and anxiety in medical students (17) and with self-reported stress experience in geriatric nurses (18).

Changes in occupational coping styles were observed following psychological interventions. One study on teachers revealed correlations between health improvement and decreases on AVEM scales *willingness to work to exhaustion*, *striving for perfection*, and the *tendency for resignation in the face of failure*, as well as with improved *distancing ability*, and *inner calm and balance* after participating in a Balint-type group intervention (19). In a randomized wait list control design, a pilot study on the effects of a mindfulness-based intervention on work-related behavior and experience patterns and depressive symptoms found that both depressive symptoms and individual attitudes such as *willingness to work to exhaustion*, *striving for perfection*, *distancing ability*, and *inner calm and balance* responded well to treatment, although overall risk patterns were less amenable to change (20).

In the current study, we investigated the responsivity of occupational behavior and experience patterns and their relationship to depressive symptom reduction in patients undergoing psychosomatic rehabilitation. Psychosomatic rehabilitation is a particular setting in that more than half of all patients may show a burnout-associated pattern (21) and are over-proportionately affected by symptom chronification and long sickness absence times (22, 23). Moreover, individuals with a burnout-associated coping style are discharged unfit for work significantly more often than individuals of any other work-related coping style (24). Using a large sample group, we therefore aimed to assess the extent of responsivity in occupational behavior and experience that may be expected in the psychosomatic rehabilitation setting. In addition, we investigated its relationship to depressive symptom reduction by assessing correlations with the BDI-II as a widely used measure of depressive symptoms. Due to previous findings (17, 18) we were particularly interested in the relationship of depressive symptom reduction and changes in the Burnout occupational coping style.

## 2 Materials and methods

The study was carried out as a multi-site correlation study in two German psychosomatic rehabilitation clinics.

### 2.1 Data acquisition

Data collection was performed as part of routine clinical diagnostics and rehabilitative interventions carried out according to German Pension Insurance (Deutsche Rentenversicherung) guidelines. Patients were asked for written broad consent on the use of clinical data for research purposes and informed about their rights to refuse data processing without indication of reasons or disadvantages to their treatment.

Approval by an ethics committee was waived in compliance with Brandenburg (§10 Datenschutz bei Forschungsvorhaben, https://bravors.brandenburg.de/de/verordnungen-215421) and Saarland (https://www.aerztekammer-saarland.de/files/164CC10E4F3/GV-0005%20Saarl%E4ndisches%20Krankenhausgesetz.pdf) state laws. A total of *N* = 1,708 psychosomatic patients (n_clinic1_ = 809, n_clinic2_ = 899) completed the self-report questionnaires Assessment of Work-related Behavior and Experience Patterns (Arbeitsbezogene Verhaltens- und Erlebensmuster, AVEM) (16) and the German version of the Beck Depression Inventory revised (BDI-II) (25) at admission (T0) and discharge (T1).

### 2.2 Measures

#### 2.2.1 AVEM

The AVEM questionnaire captures factors work engagement, psychological resilience and work-related emotions. These arise from eleven scales: (1) *work importance*, (2) *work ambition*, (3) *willingness to work to exhaustion*, (4) *striving for perfection*, (5) *ability to distance oneself*, (6) *tendency for resignation*, (7) *problem solving*, (8) *inner peace*, (9) *experience of success*, (10) *life satisfaction* and (11) *social support*. Each scale is based on six items employing a 5-level response format (“strongly agree” to “strongly disagree”) with a range between 6 and 30. The AVEM has shown good reliability throughout all scales (Cronbach’s *α* between 0.78 and 0.87; split-half reliability between 0.76 and 0.90) and stability coefficients between 0.69 and 0.82 in a 3-month period. In addition, stanine patterns in the individual scales can be used to calculate probabilities for individuals to belong to work-related coping styles G (Healthy), S (Sparing), A (Ambitious), and B (Burnout) (16).

#### 2.2.2 BDI-II

The Beck Depression Inventory (BDI-II) was developed to assess the severity of depressive symptoms. It comprises 21 questions on symptom frequency and severity within the last 2 weeks. Answers are given on a 4-level scale with response values ranging from 0 to 3. Item scores are summed up to a total score ranging from 0 to 63. The degree of depressive symptoms can be divided into four categories based on the BDI-II total score (0–13: no depression, 14–19: mild depression, 20–28: moderate depression, and 29–63: severe depression). The reliability (Cronbach’s alpha) was *α* = 0.93 in a sample of depressive patients in treatment, *α* = 0.92 in patients with primarily other mental disorders and *α* = 0.90 in a healthy population (25).

### 2.3 Data analysis

Data analysis was performed in R (Version 4.0.2) and R Studio (Version 1.3.959). Changes between T0 and T1 on AVEM scales were evaluated by t-tests for dependent samples followed by Bonferroni correction for multiple testing. Effect sizes were calculated using Cohen’s d_z_ for paired samples. Patients’ AVEM work-related coping styles at admission as well as changes in classification at discharge were determined by probability scores that reflect the likelihood of group membership in the groups of the four types G/S/A/B. The highest probability scores, which indicate profile affiliation, were counted and converted into percentages, respectively. Associations between changes in AVEM scales and probability scores of work-related coping styles with changes in depressive symptoms as measured by BDI-II were investigated by calculating the Pearson correlation of their respective change scores (T1 – T0). For all statistical tests, the *a priori* defined *α*-error level to reject the null hypothesis H_0_ was *P/P_adj._* > 0.05.

## 3 Results

### 3.1 Demographic data

Patient demographics are given in Table 1. The mean age in the sample group was 51.7 years (SD = 8.51).

**Table 1:** Demographic data.

Variable		N	%
sex	Male	540	31.6
	Female	1,168	68.4
marital status	Married	931	54.5
	Single	279	16.3
	Divorced	229	13.4
	Widowed	46	2.7
	Not specified	224	13.1
education	Special needs school / none	16	0.9
	Compulsory school	274	16.0
	High school	444	26.0
	Vocational training	781	45.7
	University	96	5.6
	Other	21	1.2
	Not specified	76	4.5
employment	Employed	1,415	82.9
	Unemployed	286	16.7
	Not specified	7	0.4
maximum duration of sick leave within last 12 months	≥ 6 months	718	42.0
	3–6 months	253	14.8
	≤ 3 months	570	33.4
	none	145	8.5
	not applicable	16	0.9
	not specified	6	0.4
sick leave (admission)	yes	948	55.5
	no	753	44.1
	not Specified	7	0.4
fit to work (discharge)	Yes	706	41.3
	No	986	57.7
	not Applicable	5	0.3
	not Specified	11	0.6
affective disorders	F32 depressive episode	417	24.4
	F33 recurrent depressive disorder	506	29.6
	F34 persistent mood disorder	45	2.6

Annotation. N = sample size.

### 3.2 Occupational behavior and experience

Statistically significant changes between T0 and T1 were observed in 8 out of 11 AVEM scales following Bonferroni correction (*work importance, work ambition, willingness to work to exhaustion*, *striving for perfection*, *ability to distance oneself*, *tendency for resignation*, *inner peace*, and *life satisfaction*). Cohen’s d_z_ effect sizes ranged from −0.45 (*striving for perfection*) to 0.43 (*ability to distance oneself*, *life satisfaction*). No statistically significant changes were observed on AVEM scales *problem solving*, *experience of success* and *social support* (Table 2).

**Table 2:** Results of paired-sample t-Tests for changes in AVEM scores between admission (T0) and discharge (T1).

AVEM scale	M_T0_ (SD)	M_T1_ (SD)	*T*	*P*	*P*_adj._	*d_z_*
1.work importance	15.74 (5.02)	14.58 (4.91)	−14.17	< 0.001	< 0.001	−0.34
2.work ambition	15.85 (4.66)	15.56 (4.65)	−3.97	< 0.001	< 0.001	−0.10
3.willingness to work to exhaustion	21.10 (5.09)	19.66 (4.92)	−16.49	< 0.001	< 0.001	−0.40
4.striving for perfection	23.64 (4.30)	22.16 (4.54)	−18.76	< 0.001	< 0.001	−0.45
5.ability to distance oneself	15.13 (5.40)	16.70 (5.38)	17.92	< 0.001	< 0.001	0.43
6.tendency for resignation	19.65 (4.86)	18.77 (4.89)	−10.65	< 0.001	< 0.001	−0.26
7.problem solving	18.88 (4.04)	18.99 (3.92)	1.51	0.132	1	0.04
8.inner peace	16.20 (4.56)	17.15 (4.41)	13.32	< 0.001	< 0.001	0.32
9.experience of success	20.11 (4.78)	20.18 (4.81)	1.03	0.301	1	0.03
10.life satisfaction	16.86 (4.77)	18.21 (4.88)	17.83	< 0.001	< 0.001	0.43
11.social support	21.01 (4.62)	21.08 (4.67)	1.01	0.311	1	0.03

Annotation. MT0 = mean at admission; MT1 = mean at discharge; SD = standard deviation; t = t-statistic; P = significance level; Padj. = adjusted significance level; dz = Cohen’s dz for repeated measures.

**Figure 1:**
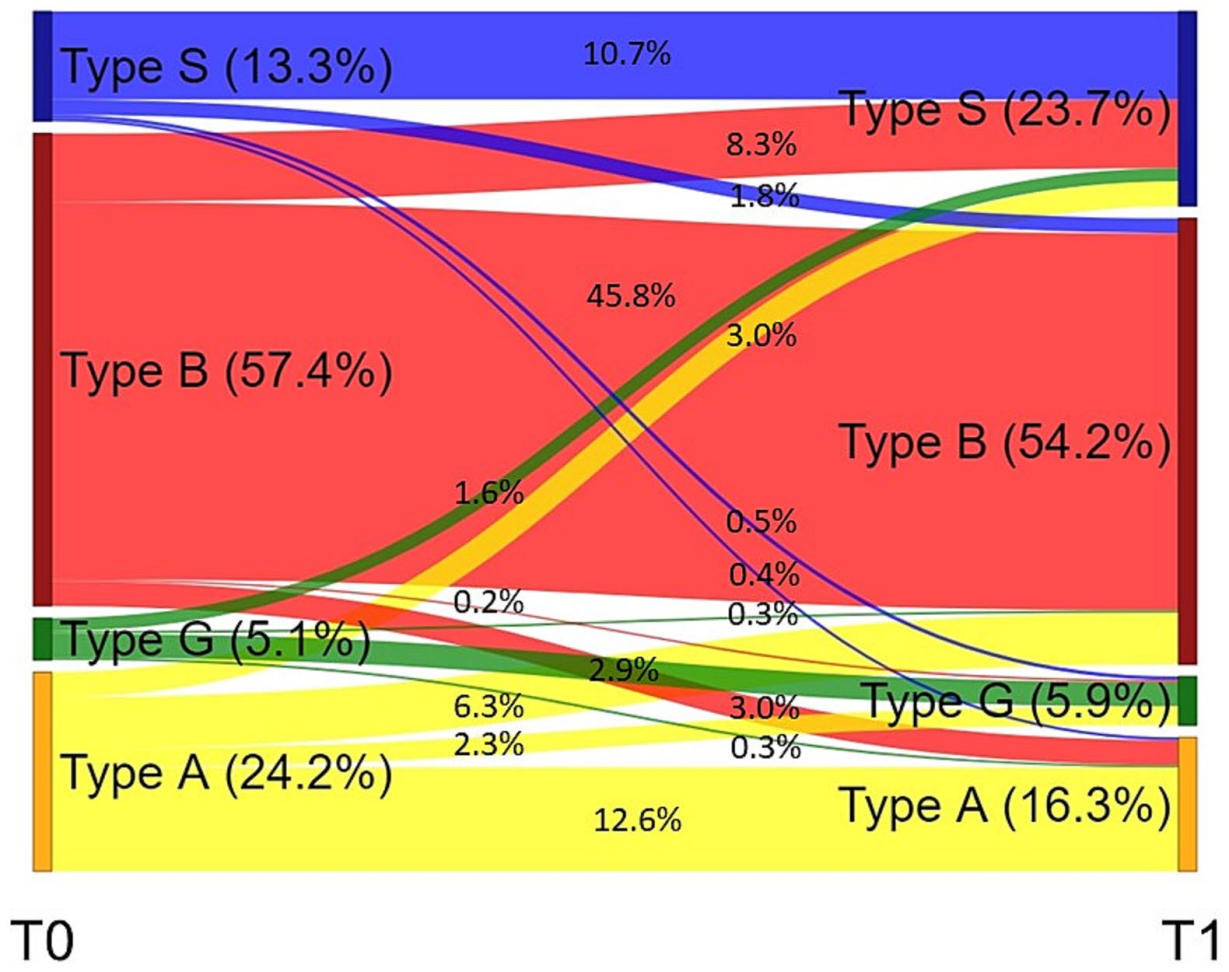
Sankey diagram of changes in occupational coping style from admission (T0) to discharge (T1). The diagram shows the percentage of patients belonging to occupational coping styles Type S (Sparing), Type B (Burnout), Type G (Healthy), and Type A (Ambitious) at admission (T0) and discharge (T1), as well as the percentage of patients changing occupational coping style between time points.

At 57.4%, the majority of psychosomatic patients presented with occupational risk pattern Type B (Burnout) at admission. The second most common pattern was risk pattern Type A (Ambitious) at 24.2%, followed by Type S (Sparing) at 13.3% and Type G (Healthy) at 5.1% (Figure 1). The highest increase (10.4%) towards discharge was observed in occupational pattern Type S, resulting in 23.7% of rehabilitants belonging to this group at T1. Out of these, 8.3% had been classified as Type B at admission. Type B remained the largest group at discharge (54.2%), with 45.8% of patients not having changed from T0, while 6.3% of scorers previously scored highest on Type A.

### 3.3 Depressive symptoms

A t-test for paired samples showed a statistically significant decrease in BDI-II scores (*t* (1707) = −46.4, *p* < 0.001*, d_z_* = −1.12) from admission (*M* = 24.7, SD = 12.0) to discharge (*M* = 13.7, SD = 12.4).

### 3.4 Association of changes in occupational behavior and experience with changes in depressive symptoms

**Figure 2:**
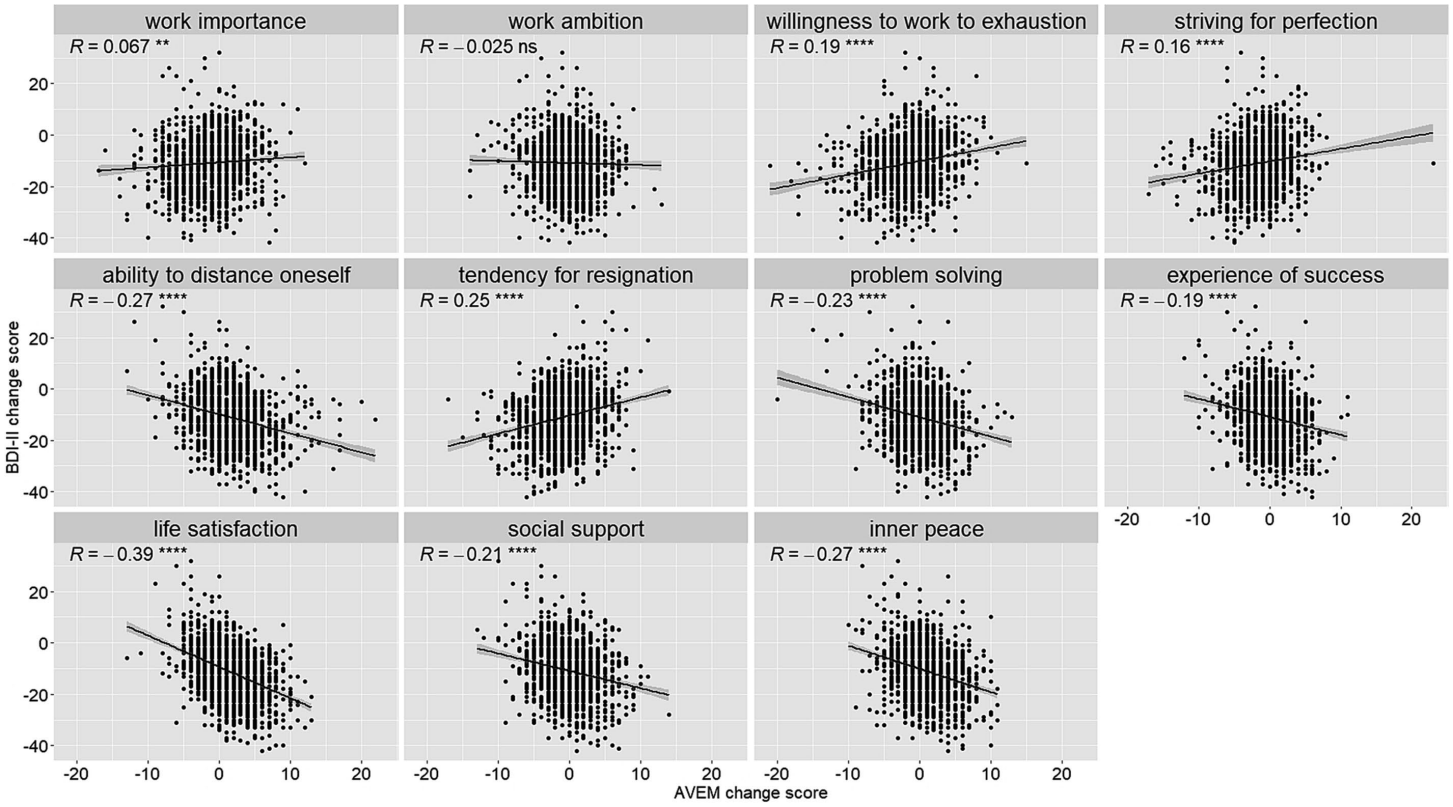
Scatter plots for AVEM scale and BDI-II change scores (T1-T0). Scatter plots show the relationship of change scores on AVEM scales with BDI-II change scores (T1-T0). Associations were determined by Pearson correlations, the level of statistical significance is indicated as ns = *p* > 0.05, ^**^ = *p* < 0.01, ^****^ = *p* < 0.0001.

Changes in BDI-II depressive symptom scores were statistically significantly associated with changes on all AVEM scales (−0.39 ≤ *r* ≤ 0.25, all *p* < 0.01) except for work ambition (Figure 2).

**Figure 3:**
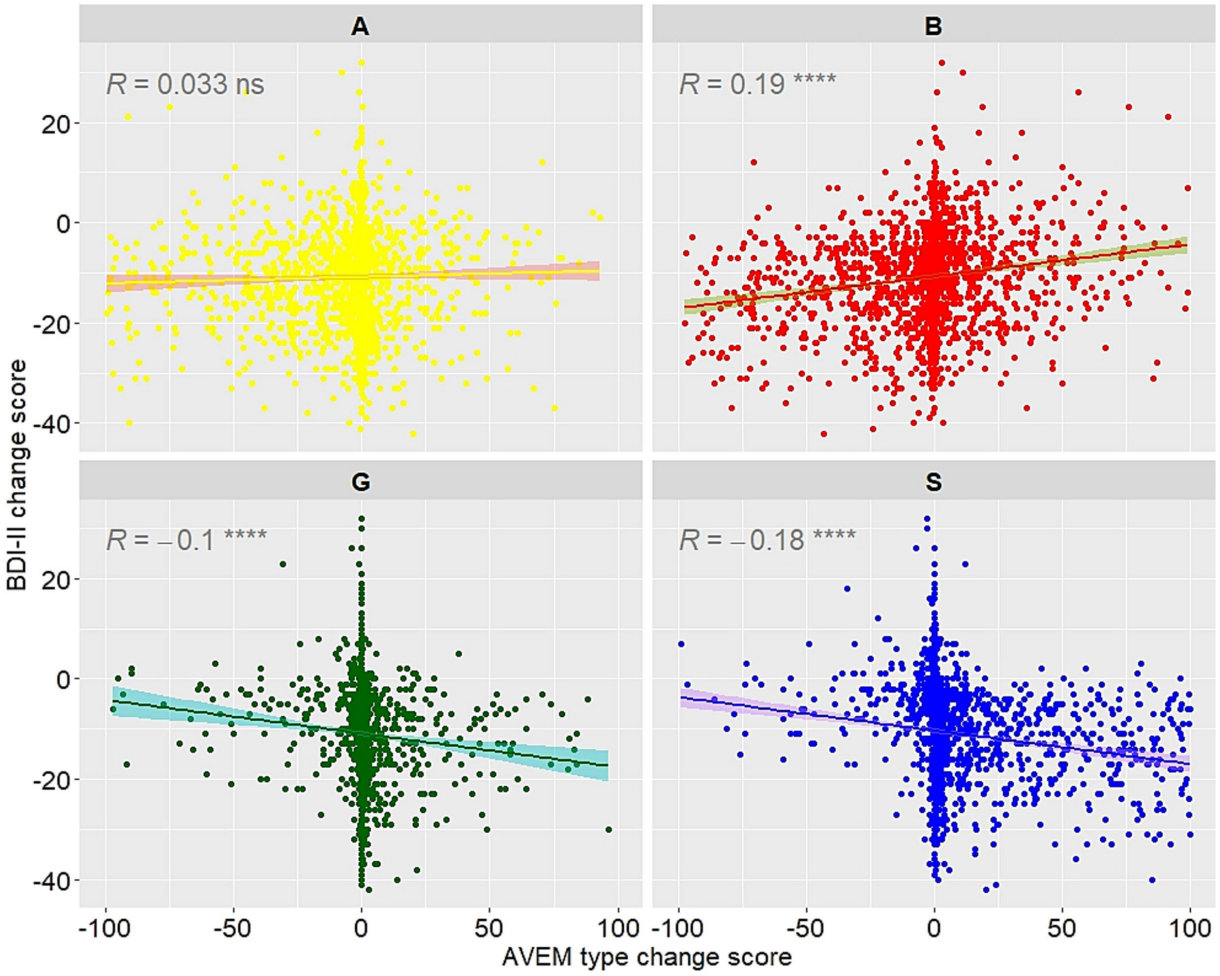
Correlation plots for AVEM work-related coping style probability change scores and BDI-II change scores (T1-T0). Scatter plots show the relationship of change scores in AVEM occupational coping style probabilities with BDI-II change scores (T1-T0). Associations were determined by Pearson correlations, the level of statistical significance is indicated as ns = *p* > 0.05, ^****^ = *p* < 0.0001.

Changes in BDI-II depressive symptoms were statistically significantly associated with changes in probability scores of all work-related coping patterns except for risk Type A (Figure 3).

## 4 Discussion

Our findings indicate that AVEM scales are moderately responsive to change in a psychosomatic rehabilitation setting, which was evident from the pre-post differences in AVEM scales with small to medium effect sizes (0.10 < Cohen’s *d_z_* < 0.50). Regarding occupational coping styles, the majority of patients were classified as Risk Type B (57.4%) and Risk Type A (24.2%) at admission. Increases towards discharge were particularly noticeable in Type S (13.3% at T0 to 23.7% at T1). Despite the highest number of transitions from one occupational coping style to another (8.3% to Type S at T1), Type B remained the largest group at discharge. In addition, changes in AVEM scales and occupational coping styles showed small to medium correlations with reductions in depressive symptoms, except for AVEM scale *work ambition* and work-related coping style Type A (Ambitious). The directions of the correlations were consistent with their underlying concepts.

While an extensive research body on the malleability of occupational experience and behavior is missing, our findings fall in line with the effects reported by previous observational and intervention studies in clinical and non-clinical populations (19, 20). Reductions in *work importance* and *work ambition* not reported in previous studies may be a result of the specific population in psychosomatic rehabilitation. Psychosomatic patients frequently present with suffering from burnout due to overinvolvement with both wage labour as well as voluntary and care work (26). While a reduction on these traits may appear counterintuitive in the context of preserving and restoring earning capacity, in this specific group a reduced focus on work is important to prevent overexertion and foster healthy behaviors such as self-care. Indeed, previous investigations showed that interventions focusing on self-care yield results superior to interventions focussing on the improvement of stress resistance (27, 28).

Interestingly, some scales were consistently unresponsive in all investigations conducted to date, i.e., *problem solving*, *experience of success* and *social support*. We assume that the psychological variables underlying these scales may indeed be inaccessible to change experience during an inpatient treatment and are more likely to manifest as secondary effects in the long run. For instance, patients less willing to work to exhaustion may learn to correct imbalances in effort and reward (29), thus eventually experiencing more satisfaction with work and life in general. In addition, lower willingness to work to exhaustion may lead to a higher propensity to delegate tasks and thereby to a higher perception of social support. Moreover, patients may simply not encounter opportunities to experience improved problem-solving skills while still in rehabilitative treatment. Future investigations may address these possibilities by including a follow-up investigation.

Correlations of changes in occupational behavior and experience patterns and depressive symptoms moreover indicated largely independent constructs and not all expression patterns on the AVEM questionnaire were associated with changes in depressive symptom load. For instance, there was no association between changes in the scale *work ambition* and depressive symptoms. The scale is highly expressed in both Risk Type A and Type G indicating that its adaptiveness in the workplace is likely to be determined by accompanying factors. That is, Risk Type A may be maladaptive due to factors such as perfectionism or the inability to distance oneself from work rather than *work ambition*.

Meanwhile, the lack in association between changes in Risk Type A and depressive symptoms over the course of rehabilitation may be due to the specific change dynamics of the profile. That is, patients scoring highest on Risk Type A at admission were equally likely to develop towards both Type S and Risk Type B at discharge. While an adaptation of Type S may conceivably result in depressive symptom relief as patients practice more emotional distancing from work, a shift towards Risk Type B may indicate the breakdown of a dysfunctional coping style laying bare the underlying exhaustion and be associated with increased depressiveness. The latter trajectory of change may seem like a deterioration, but it is important to note that this breakdown may be a necessary development for some patients to eventually improve with respect to a healthier working style and better mental health (30).

Overall, the effects reported here may give researchers and practitioners a benchmark for the extent of changes in occupational beliefs and attitudes that may be expected in the psychosomatic rehabilitation setting. While effects were moderate, it is noteworthy that around 11% of psychosomatic inpatients changed dominant coping style from Risk Types B and A to Type S during rehabilitation. Given the degree of overall chronification in the study group and limited treatment duration of 5 weeks on average, this can be considered a rather high rate. The changes in overall work attitude are furthermore likely to translate into improved work performance (31) and reduced days of sick leave (32). These implications may be of interest to employers and policy makers as mental illness is responsible for both high direct financial costs such as medication, physician services and hospitalization as well as indirect costs through losses in productivity and income (33).

The low to moderate correlations with changes in depressive symptoms indicate that occupational beliefs and attitudes and depression need to be addressed individually within the context of rehabilitation as carry-over effects may be limited. This finding is in line with the separate coding of depressive disorders and the burnout syndrome within the International Classification of Disorders (ICD), which is supported by meta-analysis (34). However, it is unclear how well the constructs are treated as separate entities in practice, which may have potentially detrimental effects on the treatment outcome. Indeed, a review on intervention practices for depression in the workplace found none of the interventions to be effective in managing depression in this context (35). Perhaps interventions implementing work-related medical rehabilitation may be better tailored to suit the needs of these patients (36).

Lastly, while changes observed in our analyses are in line with previous investigations, a limitation of our study may be that we cannot exclude confounding effects owing to the test–retest stability of the AVEM questionnaire. In addition, the setting of the study did not allow for a randomized controlled trial with an untreated control group. Moreover, treatment plans included a disorder-specific group therapy for depressive disorders, but none for the burnout syndrome. Future studies on the topic may thus utilize more in-depth diagnostics to separate the constructs and include two treatment groups, i.e., offer manualized treatments for burnout and depressive disorders within a wait list control design in order to assess the effects on treatment outcomes.

## 5 Conclusion

Occupational behavior and experience patterns showed small to medium responsivity in a psychosomatic rehabilitation setting, with a particularly noteworthy rate of transitions from Type B to Type S occupational coping styles. Changes were accompanied by small but significant associations with a reduction in depressive symptoms. Occupational beliefs and attitudes can therefore be altered during standard rehabilitative treatment and change largely independently from depressive symptoms.

## Data availability statement

The raw data supporting the conclusions of this article will be made available by the authors, without undue reservation.

## Ethics statement

Ethical approval was not required for the study involving humans in accordance with the local legislation and institutional requirements. Written informed consent to participate in this study was not required from the participants or the participants’ legal guardians/next of kin in accordance with the national legislation and the institutional requirements.

## Author contributions

LP: Conceptualization, Data curation, Formal analysis, Visualization, Writing – original draft, Writing – review & editing. CZ: Data curation, Investigation, Writing – review & editing. MK: Data curation, Investigation, Writing – review & editing, Project administration. VB: Data curation, Investigation, Validation, Writing – review & editing. VK: Conceptualization, Funding acquisition, Supervision, Writing – review & editing.
